# Supplementary material for: In-Depth Insight into the Ag/CNQDs/g-C3N4 Photocatalytic Degradation of Typical Antibiotics: Influence Factor, Mechanism and Toxicity Evaluation of Intermediates
Source: Molecules. 2023 Feb 7;28(4):1597. doi: 10.3390/molecules28041597 (PMC9962003; doi:10.3390/molecules28041597)
Supplement: Supplementary file 1 [file molecules-28-01597-s001.zip › molecules-2185854-supplementary.pdf]

# Supplementary information

for

In-depth insight into the Ag/CNQDs/g-$C_3N_4$ photocatalytic degradation of typical antibiotics: Influence factor, mechanism and toxicity evaluation of intermediates

Chen Li [1,2], Tianyi Sun [1,2,*], Guohui Yi [3], Dashuai Zhang [1,2], Yan Zhang [1,2], Xiaoxue Lin [1,2], Jinrui Liu [1,2], Zaifeng Shi [1,2,*] and Qiang Lin [1,2]

[1] Key Laboratory of Water Pollution Treatment and Resource Reuse of Hainan Province, Hainan Normal University, Haikou 571158, China
[2] School of Chemistry and Chemical Engineering, Hainan Normal University, Haikou 571158, China
[3] Public Research Laboratory, Hainan Medical University, Haikou 571199, China

Submitted to: ***Molecules***

Number of Pages: 12

Number of Texts: 4

Number of Figures: 7

*** Corresponding author: Tel. +86 13086013920; Emails: zaifengshi@163.com (Z. Shi)**

*** Corresponding author: Tel. +86 18876866664; Emails: tianyi870328@163.com (T. Sun)**

## Text S1

Synthesis of Ag/CNQDs/g-$C_3N_4$ composite

The CNQDs/g-$C_3N_4$ composites were obtained via the microwave-assisted polymerization method based on our previous literature [27]. The Ag/CNQDs/g-$C_3N_4$ composite was obtained via a modified chemical reduction method [28]. One typical procedure involves dissolving 0.2 g of CNQDs/ g-$C_3N_4$ in 200 mL of distilled water and ultrasound for 1 h. Afterwards, the suspensions was mixed with 20 mL of a solution that contained 0.59 g of sodium citrate and certain amounts of $AgNO_3$ (containing 1, 3, 5, 8 and 10 mg of silver), and then stirred the mixture 1 hour at room temperature. Following this, 20 mL of freshly prepared $NaBH_4$ (0.1 M) was added dropwise to the above mixture and stirred for 2 hours. This obtained yellowish-brown precipitate was then filtered and washed repeatedly with water before vacuum drying at 60°C overnight. The exact Ag content of different Ag/CNQDs/g-$C_3N_4$ photocatalysts powders were determined by ICP-OES. The results of ICP-OES analysis showed that the actual content of Ag in Ag/CNQDs/g-$C_3N_4$ composites with 1 wt. %, 3 wt. %, 5 wt. %, 8 wt. % and 10 wt. % were measured to be 0.94 wt. %, 2.57 wt. %, 4.68 wt. %, 8.32 wt. % and 9.63 wt. %, respectively. Besides, the photocatalytic performance of the as-prepared Ag/CNQDs/g-$C_3N_4$ composites (Ag contents: 1 wt. %, 3 wt. %, 5 wt. %, 8 wt. % and 10 wt. %) was investigated via their ability to degrade NOR (10 mg/L, 50 mL) with the 10 mg/L initial photocatalytic dosage. As shown in Fig. S1, the 3% Ag/CNQDs/g-$C_3N_4$ possessed the best photocatalytic degradation performance.

## Text S2

The main characterization procedures of photocatalysts

The photo-degradation intermediates of NOR were identified by the HPLC-MS systems at positive ion mode (Shimadzu, Japan). The specific parameter was set as follows: the capillary voltage was 3 kV and the collision voltage is 2 kV; the flow rate of desolvent gas (nitrogen) was 800 $L \cdot h^{-1}$ and the temperature was 900 °C; the cone hole voltage was 30 V; Source temperature was 600 °C; The full sweep range of the mass spectrum was 50-500 m/z. Moreover, Electron spin resonance (ESR) spectra was conducted on a Bruker model ESR EMX plus spectrometer. In the ESR detection of •OH radicals, 10 mg of Ag/CNQDs/g-$C_3N_4$ and 50 μL of DMPO were mixed uniformly in 0.5 mL deionized water and stirred for 10 min. Then the mixture was sampled by a 100 μL capillary tube and inserted into the ESR cavity immediately. As for the ESR detection of $\bullet O_2^-$, 10 mg of Ag/CNQDs/g-$C_3N_4$ and 50 μL of DMPO were mixed in 0.5 mL of $CH_3OH$ and stirred for 10 min, which was sampled as similar with the detection of •OH radicals [20]. Three-dimensional fluorescence determination of antibiotic solution before and after degradation was conducted by F-7000 fluorescence spectrophotometer (Hitachi, Japan). The specific parameter was set as follows: scanning mode was 3D-scan Fluorescence; the excitation wavelength ranges was 200-600 nm; the emission wavelength range was 200-600 nm; the excitation and emission scanning intervals were set at 10 nm; the scanning speed was 2400 nm $min^{-1}$; the slit width of excitation and emission was 5 nm. The oxford cup method was adopted to determine the microbial antibacterial activity with Escherichia coli as the reference.

## Text S3

Photocatalytic tests

Similar to our previously reported experimental methods [27], the targeted pollutant was changed to norfloxacin (NOR), Sulfamethoxazole (SMX) and Tetracycline hydrochloride (TCH) at a concentration of 10 mg/L. A 300 W Xeon-lamp equipped with a 420 nm cut-off filter was used as the visible light source ($\lambda > 420$ nm), and the light intensity was detected to be 138 ± 5 μW/cm $^2$. Typically, the photocatalyst dosage was 0.2 g/L. Notably, the suspension was kept magnetic stirring in the dark for 30 min to achieve the adsorption-desorption equilibrium. During the photocatalytic degradation reaction, 2 mL of the dispersion solution was filtrated with 0.22 μm membrane at a given time interval prior to further analysis. The high performance liquid chromatography (Shimadzu LC-20A) was quantified the residual concentration of NOR, SMX and TCH solution at 274 nm, 265 nm and 268 nm to evaluate the photocatalytic degradation effect. Moreover, the degradation intermediates were determined by the liquid chromatography-mass spectrometry (LC/MS) to further illuminate the mechanism of photocatalytic degradation of NOR. In addition, the effects of initial pH, coexistence of humic acid (HA) and anion-cation were also investigated.

## Text S4

Theoretical calculation

Based on the density functional theory (DFT), all calculations were carried out using the Gaussian 03 package, and the geometric optimizations were performed with the hybrid B3LYP method at 6-31(d, p) basis set level. There has been extensive use of Fukui functions as a way to predict the reaction sites for electrophilic, nucleophilic, and free radical attacks. Specifically, Fukui function is defined as:

$$f(r) = \left(\frac{\partial^2 E}{\partial N.\partial v(r)}\right) = \left[\frac{\partial \mu}{\partial v(r)}\right]_N = \left[\frac{\partial \rho(r)}{\partial N}\right]_{V(r)} \quad (1)$$

Where ρ(r) was the electron density at the point R in space, N was the number of electrons in the existing system, and the constant term v in the partial derivative was the external potential. In Fukui function calculations, the atomic population was used to indicate the number of electron density distributions around an atom. Fukui functions could be explicitly calculated in the following three cases:

Nucleophilic attack: $f_k^+ = q_N^k - q_{N+1}^k$ (2)

Electrophilic attack: $f_k^- = q_{N-1}^k - q_N^k$ (3)

Free radical attack: $f_k^0 = (q_{N-1}^k - q_{N+1}^k)/2$ (4)

Where $q^k$ was the atomic charge population of k atom in the corresponding state. Fukui function contains information about different sites of a molecule, and the exact reaction site of the molecule was usually larger than the reduced Fukui function (CFF) value of other regions. In this study, $\bullet O_2^-$ was the main attacking group, so free radical attacking CFF was used to analyze the regional selectivity of NOR, SMX and TCH degradation.

# Figures

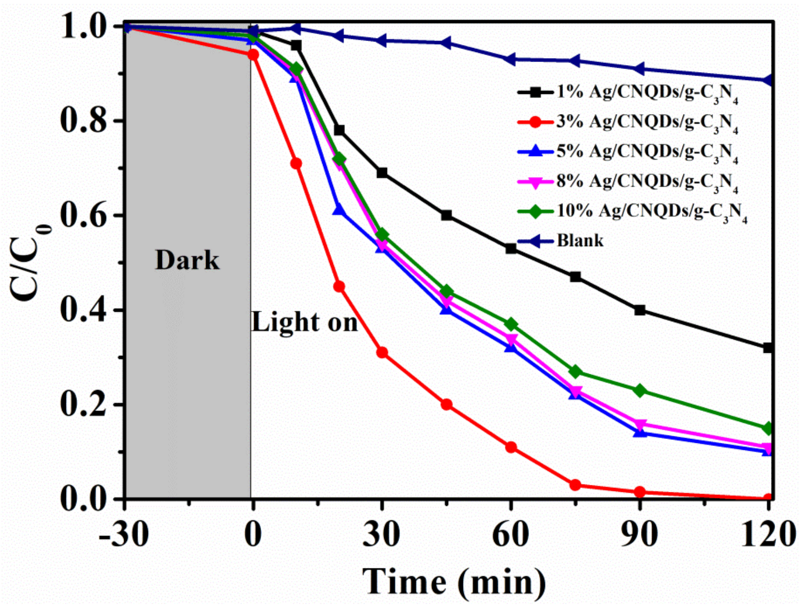


**Figure S1** Ag content optimization.

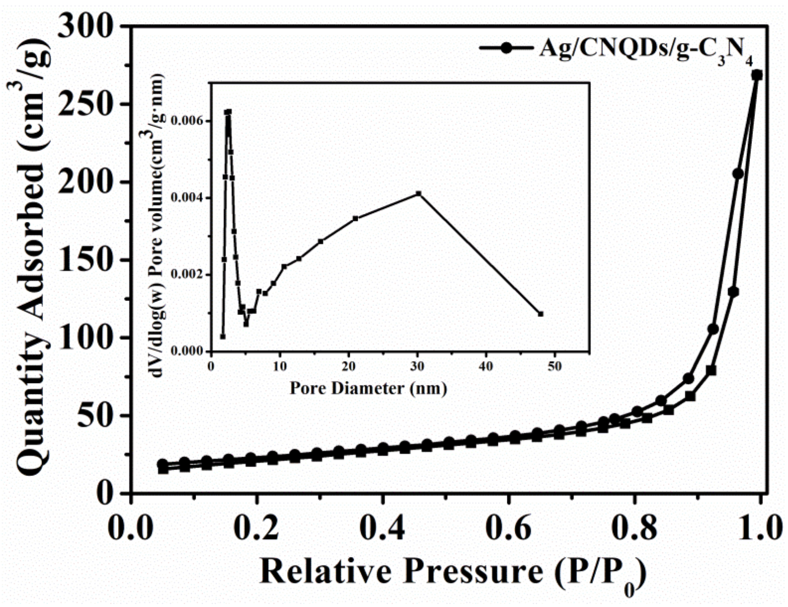


**Figure S2** Nitrogen adsorption-desorption isotherms of samples.

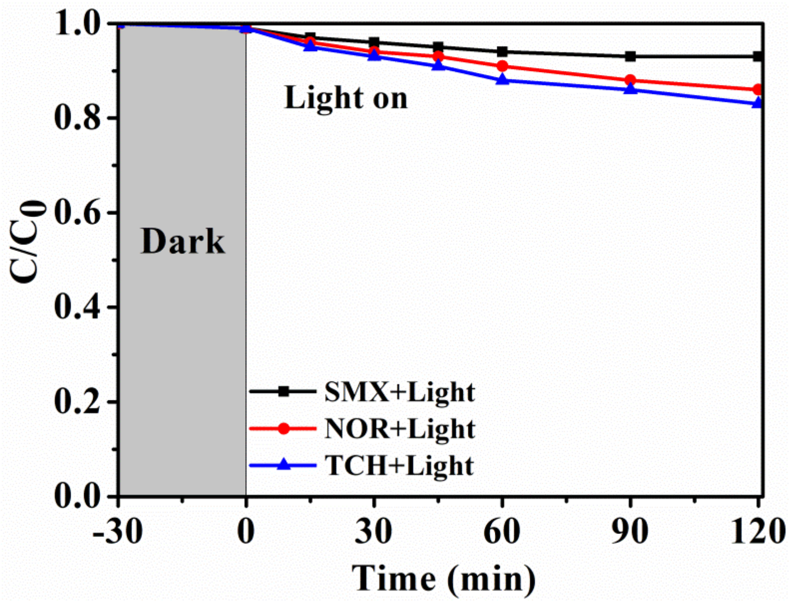


**Figure S3** Photo-degradation curves of NOR, SMX and TCH under the condition of light irradiation without photocatalyst.

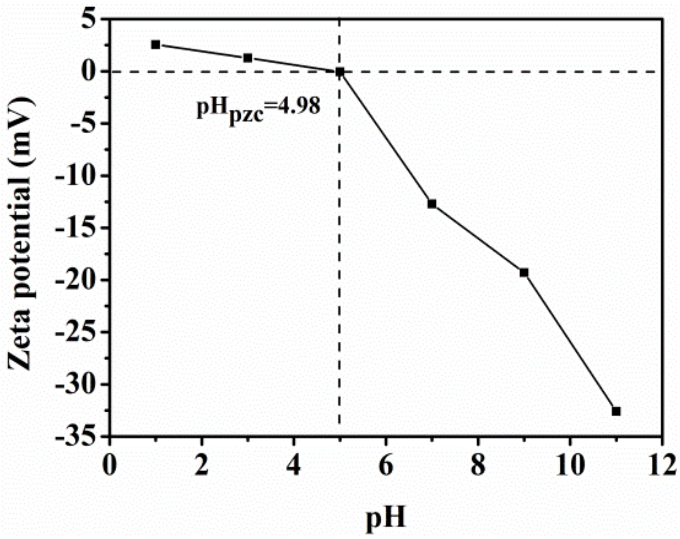


**Figure S4** Surface Zeta potential of Ag/CNQDs/g-$C_3N_4$.

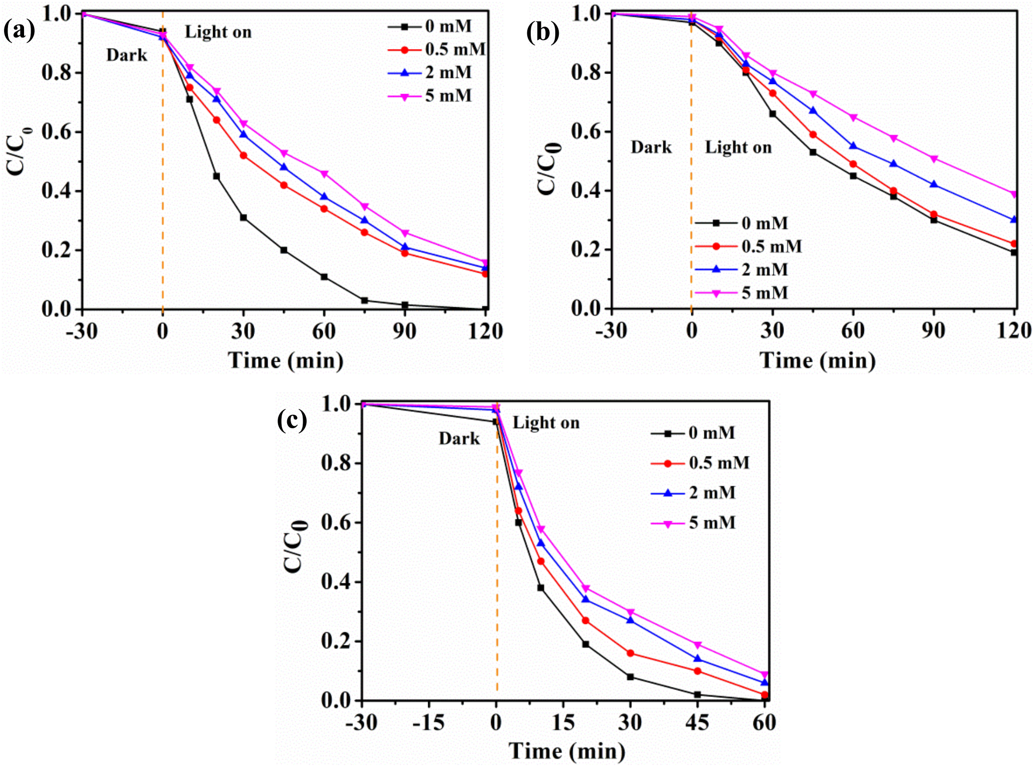


**Figure S5** Effect of $Cl^-$ on the degradation efficiency of (a) NOR, (b) SMX and (c) TCH by Ag/CNQDs/g-$C_3N_4$ (Conditions: antibiotic concentration = 10 mg $L^{-1}$, [catalyst] = 0.2 g $L^{-1}$, reaction temperature = 25 ℃).

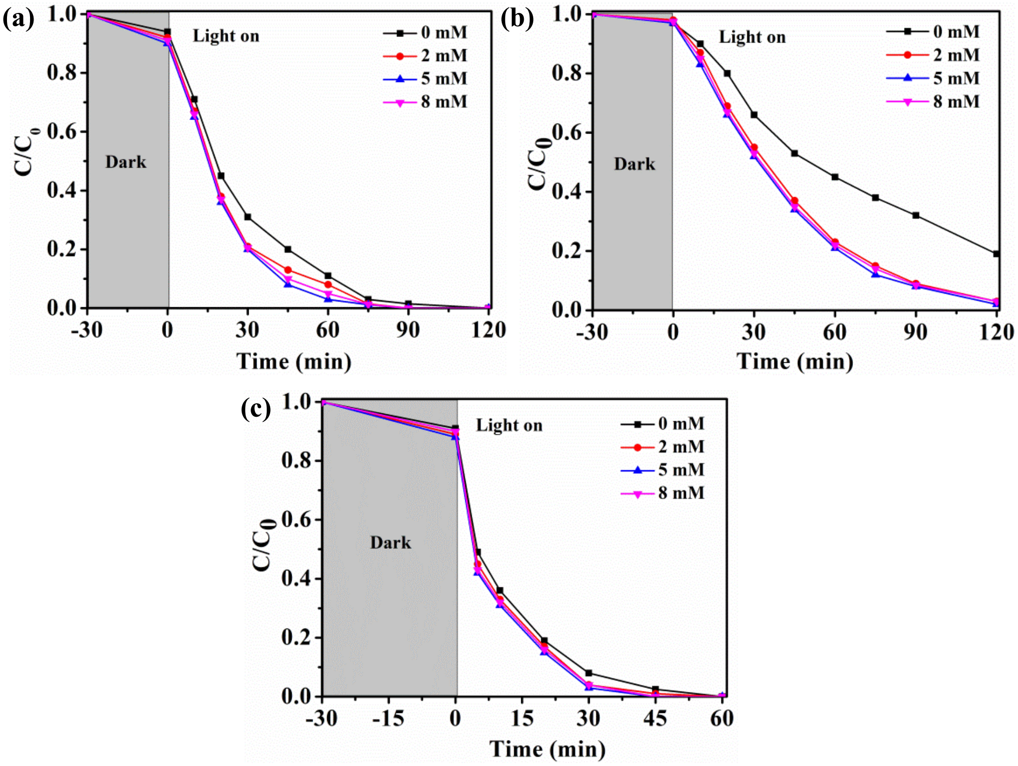


**Figure S6** Effect of $HCO_3^-$ on the degradation efficiency of (a) NOR, (b) SMX and (c) TCH by Ag/CNQDs/g-$C_3N_4$ (Conditions: antibiotic concentration = 10 mg $L^{-1}$, [catalyst] = 0.2 g $L^{-1}$, reaction temperature = 25 ◦ C).

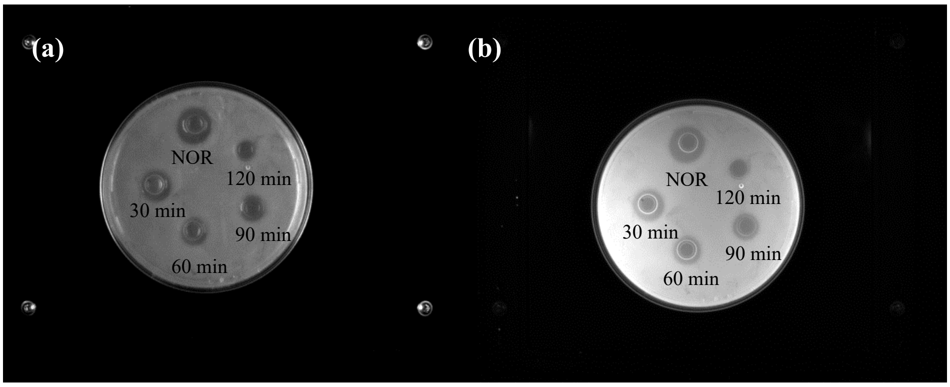


**Figure S7** Antibacterial activities of NOR (a) with only light irradiation and (b) only photocatalyst treatment.
